# The Effect of Age on Improvements in Vestibulo-Ocular Reflexes and Balance Control after Acute Unilateral Peripheral Vestibular Loss

**DOI:** 10.3389/fneur.2016.00018

**Published:** 2016-02-18

**Authors:** Alja Scheltinga, Flurin Honegger, Dionne P. H. Timmermans, John H. J. Allum

**Affiliations:** ^1^Division of Audiology and Neurootology, Department of ORL, University Hospital of Basel, Basel, Switzerland; ^2^Radboud University, Nijmegen, Netherlands

**Keywords:** vestibular loss, aging, vestibulo-ocular reflex, vestibulo-spinal reflex, balance control

## Abstract

**Background:**

An acute unilateral peripheral vestibular loss (aUVL) initially causes severe gaze and balance control problems. However, vestibulo-ocular reflexes (VOR) and balance control are nearly normal 3 months later as a result of peripheral recovery and/or central compensation. As pre-existing vestibular sensory loss is assumed to be greater in the healthy elderly, this study investigated whether improvements in VOR and balance function over time after aUVL are different for the elderly than for the young.

**Methods:**

Thirty aUVL patients divided into three age-groups were studied (8 age range 23–35, 10 with range 43–58, and 12 with range 60–74 years). To measure VOR function eye movements were recorded during caloric irrigation, rotating chair (ROT), and head impulse tests. Balance control during stance and gait was recorded as lower trunk angular velocity in the pitch and roll planes. Measurements were taken at deficit onset, and 3, 6, and 13 weeks later.

**Results:**

There was one difference in VOR improvements over time between the age-groups: Low acceleration ROT responses were less at onset in the elderly group. Deficit side VOR responses and asymmetries in each group improved to within ranges of healthy controls at 13 weeks. Trunk sway of the elderly was greater for stance and gait at onset when compared to healthy age-matched controls and the young and greater than that of the young and controls during gait tasks at 13 weeks. The sway of the young was not different from controls at either time point. Balance control for the elderly improved slower than for the young.

**Conclusion:**

These results indicate that VOR improvement after an aUVL does not differ with age, except for low accelerations. Recovery rates are different between age-groups for balance control tests. Balance control in the elderly is more abnormal at aUVL onset for stance and gait tasks with the gait abnormalities remaining after 13 weeks. Thus, we conclude that balance control in the elderly is more affected by the UVL than for the young, and the young overcome balance deficits more rapidly. These differences with age should be taken into account when planning rehabilitation.

## Introduction

Because of its ability to detect linear and angular body accelerations, the vestibular system plays a crucial role in static and dynamic balance control (1). This role includes stabilizing the head and trunk, especially on unstable surfaces (2). Following acute unilateral vestibular loss (aUVL) due to acute vestibular neuritis or following eight nerve neurectomy, vestibular signals driving vestibulo-ocular reflexes (VOR) and vestibule-spinal reflexes (VSR) are inaccurate or absent causing postural instability (3, 4). Effects of aUVL on the VOR include spontaneous nystagmus, skew deviation, eye cyclotorsion, VOR gain reductions, and phase changes (1, 5, 6). VSR contributions to balance control are also affected. Head and body tilt and deviation of the locomotor trajectory toward the affected side as well as stance and gait instability have been observed as a result (5, 7, 8).

Patients with aUVL can regain normal gaze and balance control after an aUVL (7, 9–11) but the extent to which patients recover normal function can differ between patients (1) and between stance and gait tasks (4). Recovery of function can occur via peripheral vestibular recovery and/or central compensation (3). However, the recovery rates for VOR and VSR measures (as recorded during balance control tasks) differ. Furthermore, VOR and balance control measures are weakly correlated with one another (4, 11). Whether differences in recovery rates of VOR and balance measures differ with age is not known.

As with other sensory systems, e.g., hearing, the vestibular sensory system deteriorates with age (12–14). Because vestibular reflexes contribute to both the gaze and balance control, both functions are assumed to decline with age, too (12, 15). The elderly (those over 60 years of age) have greater trunk angular sway during stance and stance tasks compared to younger subjects (16–19). However, weak correlations between changes in VOR function and balance control as a result of subject age have been found (20). Thus, despite the decline in the numbers of hair cells in the peripheral vestibular system with age (21), VOR function does not decline with age to the same extent as the decline in balance control during stance (18, 22–24). Given this difference between how VOR and balance control declines with age the question arises if, with an aUVL, balance control is changed more compared to the VOR in the elderly than in the young. A difference would have important clinical implications, because clinically it is often assumed that VOR and balance control functions are correlated (9).

Currently it is not known if the improvements in VOR and VSR function (based on measures of balance control) over time following an aUVL are different between the elderly and the young. This study therefore focused on the extent to which age might affect improvement of VOR and VSR function in aUVL patients. This knowledge could be useful when establishing evidence-based therapy regimes appropriate for the young and elderly following an acute UVL.

## Materials and Methods

### Subjects

Consecutively collected patient data from the University Hospital Basel was examined retrospectively for this study approved by the ethical committee of NW Switzerland (EKNZ). The 30 subjects (13 females and 16 males) with an aUVL diagnosed as presumably vestibular neuritis on the basis of a pathological canal paresis, the presence of a spontaneous nystagmus beating toward the healthy ear, nausea, and the constant presence of symptoms over hours were subdivided into the following three groups: young (<35 years), middle-aged (36 < years < 60), and elderly (>60 years). Patients were excluded from this study if they had a previous history of balance problems related to the inner ear or had concurrent neurological or orthopedic problems affecting balance. The young group consisted of four men and four women with a mean age of 28.1 years (range 23–35). The middle-aged group consisted of six men and four women with a mean age of 51.4 years (range 43–58). The elderly group consisted of six men and six women with a mean age of 65.7 years (range 60–74). Measurements were taken at acute onset of the UVL (within 2–5 days of the patient’s entry into in-patient hospital care), and planned for 3, 6, and 12 weeks after onset. Average times were, however, 3, 6.2, and 13.1 weeks. Although all subjects were measured at onset, not every subject could be measured four times, which resulted in 27 subjects being measured at 3 weeks; 25 subjects at 6.2 weeks; and 25 subjects at 13 weeks. All patients were treated intravenously with methylprednisolone (125 mg Solumedrol™ per day) and then discharged 4 days after entry as an in-patient with oral medication. On discharge, patients were offered 10 sessions of balance-oriented physical therapy. Apart from comparisons between patient group means over time, group data was compared with that of an equal number of age-matched healthy controls recorded previously (6, 7, 18, 25). Written informed consent was obtained from the patients to use their data anonymously.

### Measurement Systems

To measure VOR function in response to high accelerations (above 2000°/s^2^), one of two video Head Impulse Test (vHIT) systems was used [ICS system from GN Otometrics and EyeSeeCam (ESC) from Interacoustics]. Both systems were used according to the protocol described by MacDougall et al. (26) with head velocities reaching 100–250°/s by 100 ms. At least 20 head rotations to each side were performed. During the head movements, the patient was seated and fixed gaze on a small target 3 m away. Sections of the data with covert saccades and artifacts are removed from the recordings prior to gain calculations by the vHIT manufacturer’s software. Gains were calculated based on the quotient of the areas under the eye and head velocity impulse responses for the ICS system. The interval used started 100 ms prior to peak head velocity and ended when head velocity first crossed zero after the peak. For the ESC system, a regression between eye and head velocity was performed over the first 100 ms of data following the onset of head velocity defined as first exceeding 20°/s. As the ICS and ESC methods do not yield the same gain values (the regression fit yields lower gain values), we corrected ESC gain values to equivalent ICS gains based on quadratic fit between the gain values obtained from the two methods (27).

Rotating Chair tests (ROT) were performed according to the previous descriptions (25, 28). The ROT was performed with low accelerations of 20 and 5°/s^2^. For these tests, horizontal whole body rotation was performed in darkness using periods of constant acceleration reaching velocities of 120 and 200°/s, respectively. Subjects were seated in the rotating chair (Tönnies, Wurzburg) with the head fixed to the chair. A triangular velocity profile was used for the 20°/s^2^ acceleration and slow phase eye velocity (SPV) amplitude of the nystagmic eye movements was measured at its peak just after the chair reached a velocity of 120°/s (28). For the constant 5°/s^2^ acceleration over 40 s to a velocity of 200°/s the mean level of SPV between 30 and 40 s of acceleration was used as the VOR measure (25). Further details of these vHIT and ROT tests are described in Allum and Honegger (11).

A SwayStar™ system (Balance International Innovations, Switzerland) was used to measure balance control and thereby VSR contributions to balance control. This system was attached to the trunk at L1–3 using a converted motor-cycle belt. The gyroscopes systems measured angular velocity in the pitch and roll planes from which angular displacements were calculated with trapezoid integration on-line. The same standard protocol of 14 stance and gait tasks was used as described before to measure balance control (7). Tasks were performed by the participants without shoes. Stance tasks consisted of standing on one and two legs with eyes open and closed. All stance tasks were ended after 20 s or when the participant lost balance or the non-stance foot touched the ground. Standing on one leg trials were performed on the preferred leg. All stance tasks except the standing on one leg eyes closed trial were also repeated on a foam support surface (thickness 10 cm, width 50 cm, length 150 cm, and density 25 kg/m^2^). A semi-stance gait-like task, walking eight tandem steps, was performed on a normal floor and on the foam support system with the participants observing their feet while walking. Five gait tasks were all performed at the subjects’ preferred gait speed. Three consisted of walking 3 m with either eyes closed, while rotating the head left and right or while pitching the head up and down. The fourth gait task was to walk over four low barriers, each 24 cm high spaced 1 m apart. The final task was to walk up and down a set of stairs consisting of two up- and two downward steps, each 23 cm high. During all trials one or two spotters, as necessary, stood next to the participant to prevent a fall in case of loss of balance. The duration of each gait trial was the time needed to complete the task or to when the subject lost balance. As measures of balance control we used the peak-to-peak angular displacement and velocity in the roll and pitch directions (see lower right Figure 2) from each trial as well as trial durations.

### Data Analysis

A Wilcoxon signed rank test was performed to determine if there were differences in VOR and balance measures over time. A Kruskall–Wallis test was performed with the age-group as grouping variable to calculate whether test results differed for different age-groups and to show if there was any effect of age on recovery between onset of the aUVL and at 13 weeks later.

The mean recovery time (as well as of the mean plus and minus the SEM) of balance measures was modeled by the following equation, $y=p_{1}+p_{2}\cdot e^{-p_{3}t}$, where *y* is the measured mean at time, *t*, in weeks, *p*_1_ the steady state mean value of the measure, *p*_2_ the difference between onset and steady state means, and 1/*p*_3_ the exponential decay time constant of the mean between onset and steady state. The parameters of the exponential model function were estimated using MATLAB’s *nlinfit* (non-linear least-squares regression) function. Further details may be obtained from Allum and Honegger (4).

## Results

### Effect of Age on VOR at Onset of an aUVL and 13 Weeks Later

**Figure 1:**
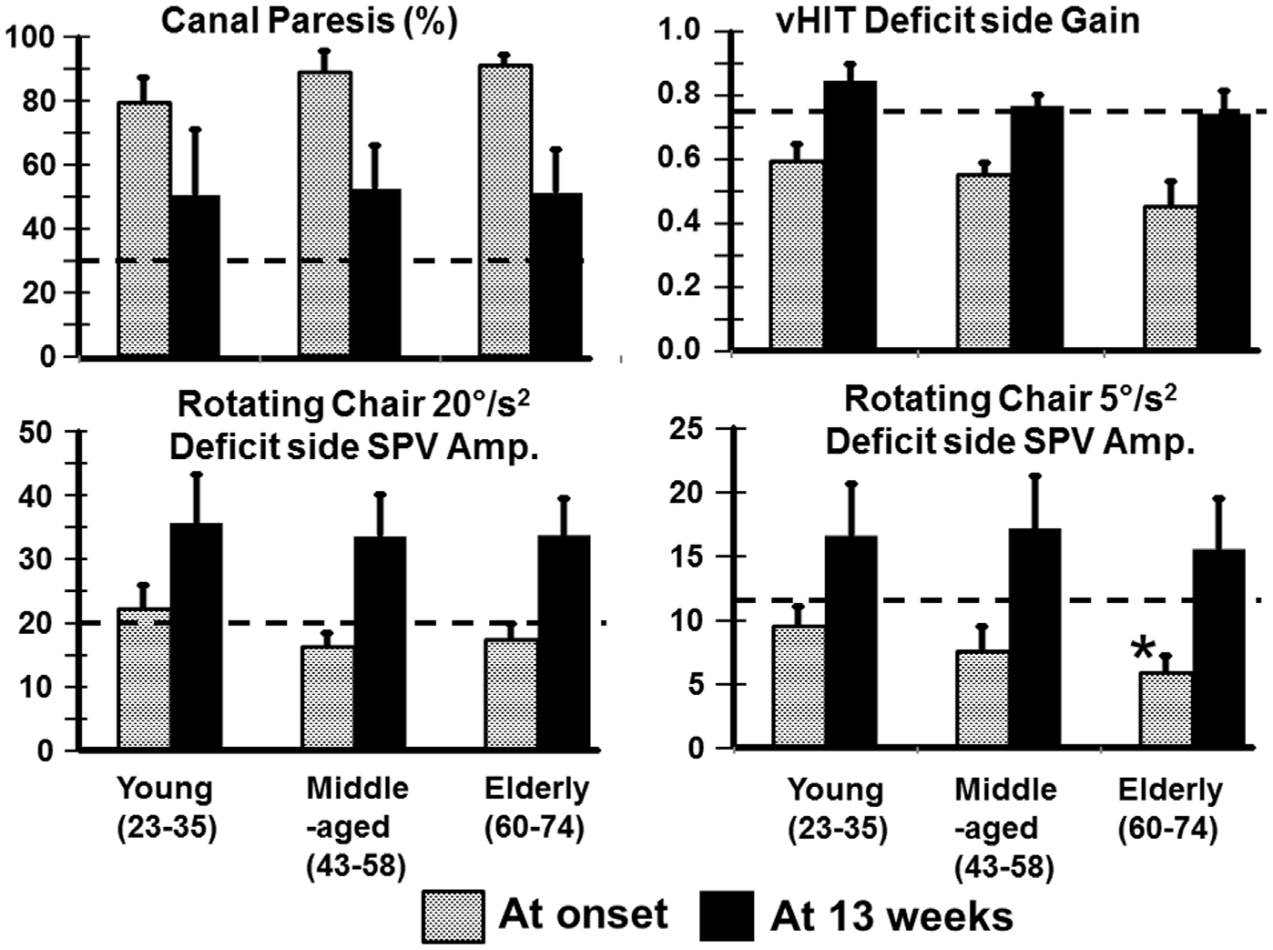
**Means of VOR values (and SEM) for young, middle-aged, and elderly patients recorded at onset and 13 weeks after an aUVL**. The column heights represent the mean value and the vertical bars on the columns the SEM. The horizontal dashed line marks the upper (for canal paresis) and lower (for responses to heads rotation to the deficit side) 95% limits of healthy normal subjects. The asterisk marks a value for the elderly significantly different from the young (*p* < 0.05).

Most VOR measures were not different between the age-groups. For example, the level of spontaneous nystagmus as determined by its SPV was not different between the groups at onset of the aUVL or 13 weeks later. The mean levels for the young, middle-aged, and elderly were 7.4, 10.3, and 6.3°/s with SDs of 6.1, 5.7, and 3.1, respectively. At 13 weeks, the level of spontaneous nystagmus was 1.3, 1.9, and 0.8°/s with SDs of 1.7, 2.0 and 0.7°/s, respectively. There were slightly more deficits on the right side for young (61.5%) compared to the elderly (41.6%). The middle-aged had 40% of the deficits on the right. In addition, there were almost no significant differences between the age-groups for mean VOR responses after aUVL. At aUVL onset, VOR deficit side values were outside of the range of healthy normal as indicated by the 95% limits shown in Figure 1, and the statistics of Table 1; likewise for the corresponding asymmetries. The latter were greater than normal across all age-groups at aUVL onset. At 13 weeks, mean VOR responses for each group were in the normal range with the exception of the canal paresis values from the caloric test which remained, on average, greater than the normal upper limit of 30% for all groups.

**Table 1:** **Significant (*p*) comparisons at onset of an aUVL and 13 weeks later with respect to twice the number of healthy age-matched normal subjects for young (23–35 years of age *N* = 8) and elderly (60–74 years of age, *N* = 12) UVL patients**.

VOR test	Measure	Young at aUVL onset	Young after 13 weeks	Elderly at aUVL onset	Elderly after 13 weeks
Caloric	CP%	**0.000**	ns	**0.000**	**0.009**
vHIT	Def side	**0.047**	ns	**0.013**	ns
ROT 20°/s^2^	Def side	**0.000**	ns	**0.000**	ns
	Asymm	**0.001**	ns	**0.000**	ns
ROT 5°/s^2^	Def side	**0.000**	ns	**0.000**	ns
	Asymm	**0.000**	ns	**0.001**	ns
**Balance Test**	**Measure**	**Young at aUVL onset**	**Young after 13 weeks**	**Elderly at aUVL onset**	**Elderly after 13 weeks**

s2ecf	Pivel	ns	ns	**0.01**	ns
	Rovel	ns	ns	**0.019**	ns
w8tan	Pivel	ns	ns	**0.014**	ns
	Rovel	ns	ns	**0.000**	**0.000**
	Piang	ns	ns	**0.000**	0.056
	Roang	ns	ns	**0.000**	**0.006**
	Dur	ns	ns	**0.006**	ns
w3mhp	Pivel	ns	ns	**0.021**	ns
	Rovel	ns	ns	**0.004**	ns
	Piang	ns	ns	**0.048**	**0.044**
	Roang	ns	ns	**0.037**	ns
	Dur	ns	**0.013**	ns	**0.011**
w3mec	Pivel	ns	ns	**0.003**	ns
	Rovel	ns	ns	**0.026**	ns
	Piang	ns	ns	ns	ns
	Roang	ns	ns	**0.036**	**0.023**
	Dur	ns	**0.006**	ns	ns

*Mean and SEM for the listed measures are provided in the figures*.

*In the upper part of the table of VOR measures CP stands for canal paresis; vHIT, video head impulse test; ROT 20°/s^2^, rotating chair test with 20°/s^2^ acceleration; ROT 5°/s^2^, rotating chair test with 5°/s^2^ acceleration. Def side stands for deficit side gain for vHIT and slow phase velocity peak amplitudes for the ROT test, Asymm for response asymmetry*.

*In the lower part of the table listing balance tests s2ecf stands for standing on two legs on foam with eyes closed; w8tan, walking eight tandem steps; w3mhp, walking 3 m while pitching the head up and down; w3mec, walking 3 m with eyes closed. ns for not significant*.

*Values significantly greater than normal reference values are marked in black bold font, those less, in gray font and underlined*.

Relatively more of the elderly had no improvement of peripheral vestibular function at 13 weeks (42% with a CP remaining greater than 90% compared with 25% young). However, the number of patients with full peripheral recovery (CP <30%) at 13 weeks was the same across the groups (50% of young, 40% of middle-aged, and 33% of elderly). Figure 1 also shows that generally differences in deficit side responses between onset and 13 weeks were not present between the age-groups although there was a weak trend for decreases in VOR deficit side responses with age. This trend was only significant for normal (*p* = 0.007) and deficit side (*p* = 0.031) responses of the elderly with respect to the young for 5°/s ROT responses at aUVL onset (Figure 1). In keeping with this trend, a larger number of the elderly had ROT responses to the deficit side, which were not compensated to the lower 5% bound of normal levels (20.1°/s for 20°/s^2^ and 11.5°/s for 5°/s^2^ rotations at 13 weeks). That is 12% of the young, 0% of the middle-aged, but 33% of the elderly were not compensated for 20°/s^2^ ROT at 13 weeks. The corresponding figures for 5°/s2 ROT were 12, 30, and 42%, respectively.

### Effect of Age on Balance Tests at Onset of an aUVL and 13 Weeks Later

**Figure 2:**
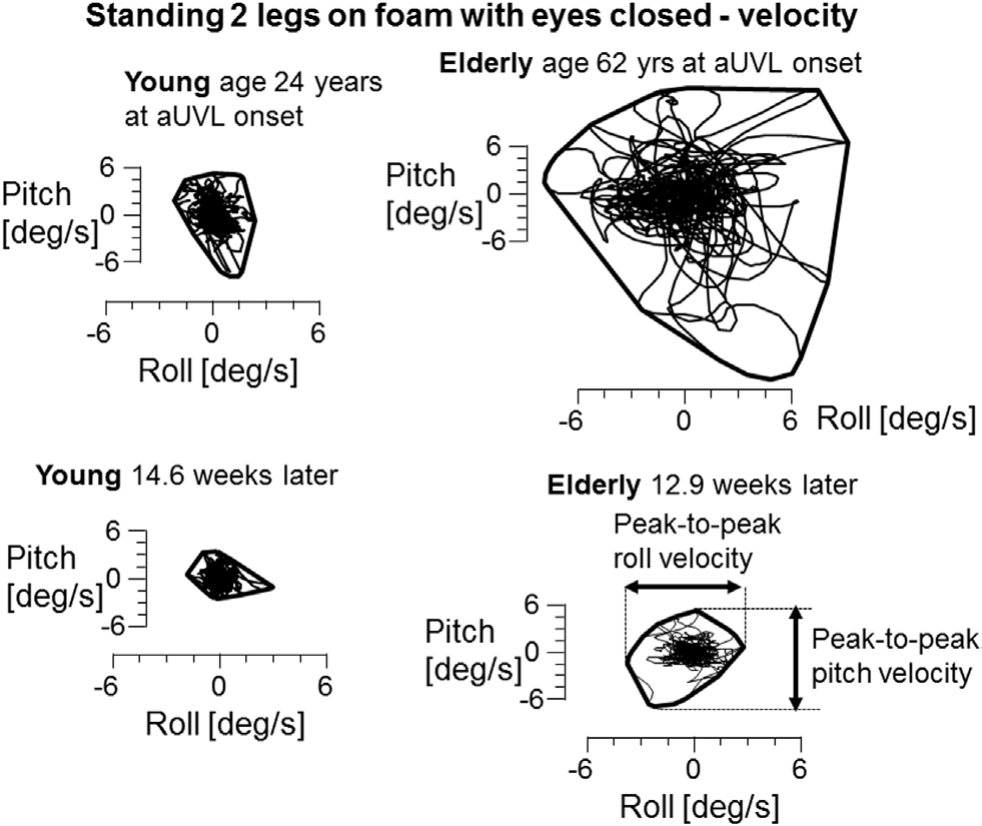
***x*–*y* plots of trunk sway roll versus pitch angular velocity during the task of standing 2 legs on foam with eyes closed**. Both the young and elderly patients in the plots had no peripheral vestibular recovery. CP changed from 84 to 92% for the elderly patient and from 85 to 95% for the young patient. A convex hull has been plotted around each set of traces to show the limits of sway. The measures used to calculate differences in angular velocities between the groups are marked in the lower right *x*–*y* diagram.

**Figure 3:**
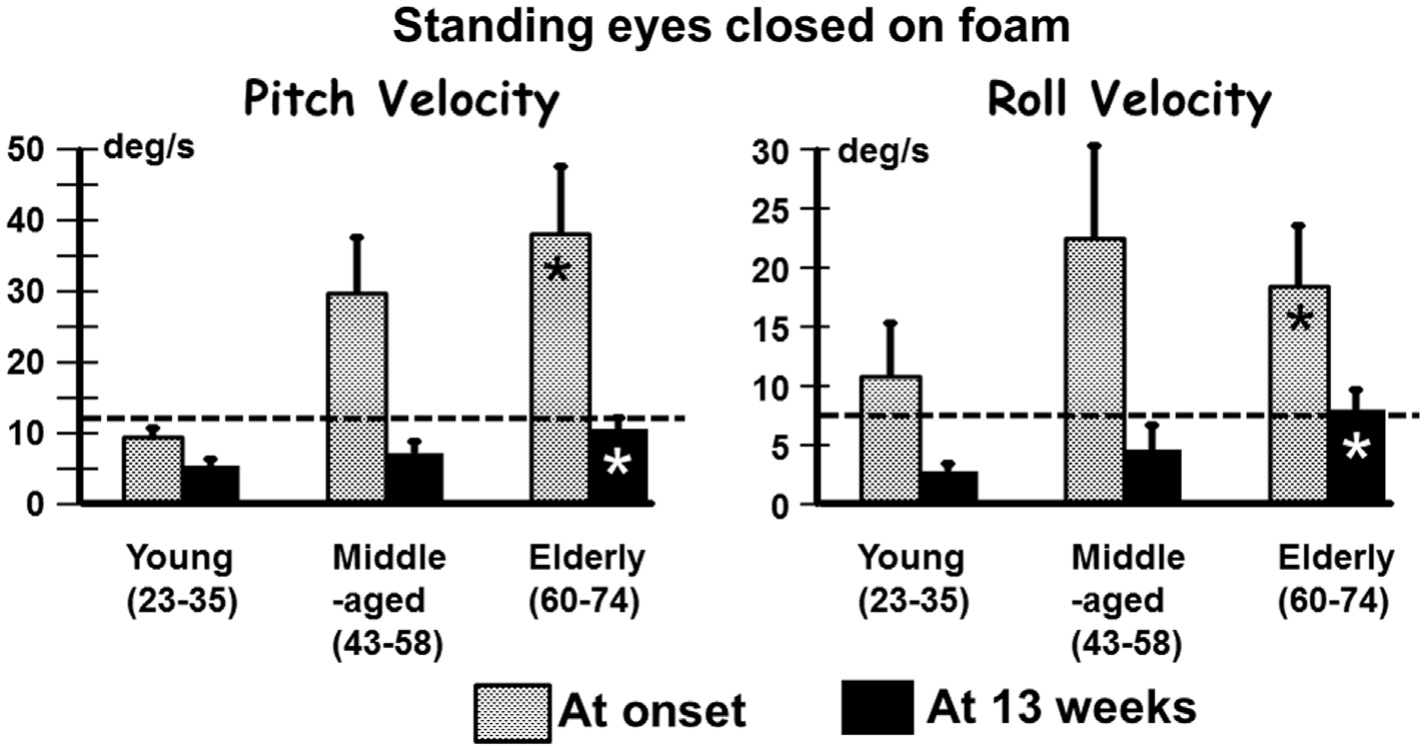
**Means (and SEMs) of pitch and roll velocity for the task of standing eyes closed on foam**. The layout of the figure is identical to that of Figure 1.

In contrast to the limited number of differences with age for VOR responses, we observed several differences for stance and gait tests, specifically for those balance tests typically pathological for aUVL patients (4, 7). Figure 2 shows an example of the differences for the stance test standing on two legs eyes closed on foam. In Figure 2, the original recordings are transformed into *x*–*y* (roll versus pitch) velocity plots, which depict the differences between the elderly and the young more clearly. Both patients in the figure had no peripheral vestibular recovery so that any improvement in balance function could only be due to central compensation. The appearance in Figure 2 of the elderly having greater sway at aUVL onset and 13 weeks later is confirmed (*p* < 0.03) by the columns marked with asterisks in Figure 3. As also indicated by Figure 3 and Table 1, pitch and roll velocity values were outside the normal range across all three age populations at onset except for pitch velocity values for the young. The greater sway at aUVL onset in the elderly was the main reason why the improvement in pitch velocity over 13 weeks was significantly greater (*p* = 0.05) in the elderly. That is, there was a greater possibility for improvement.

**Figure 4:**
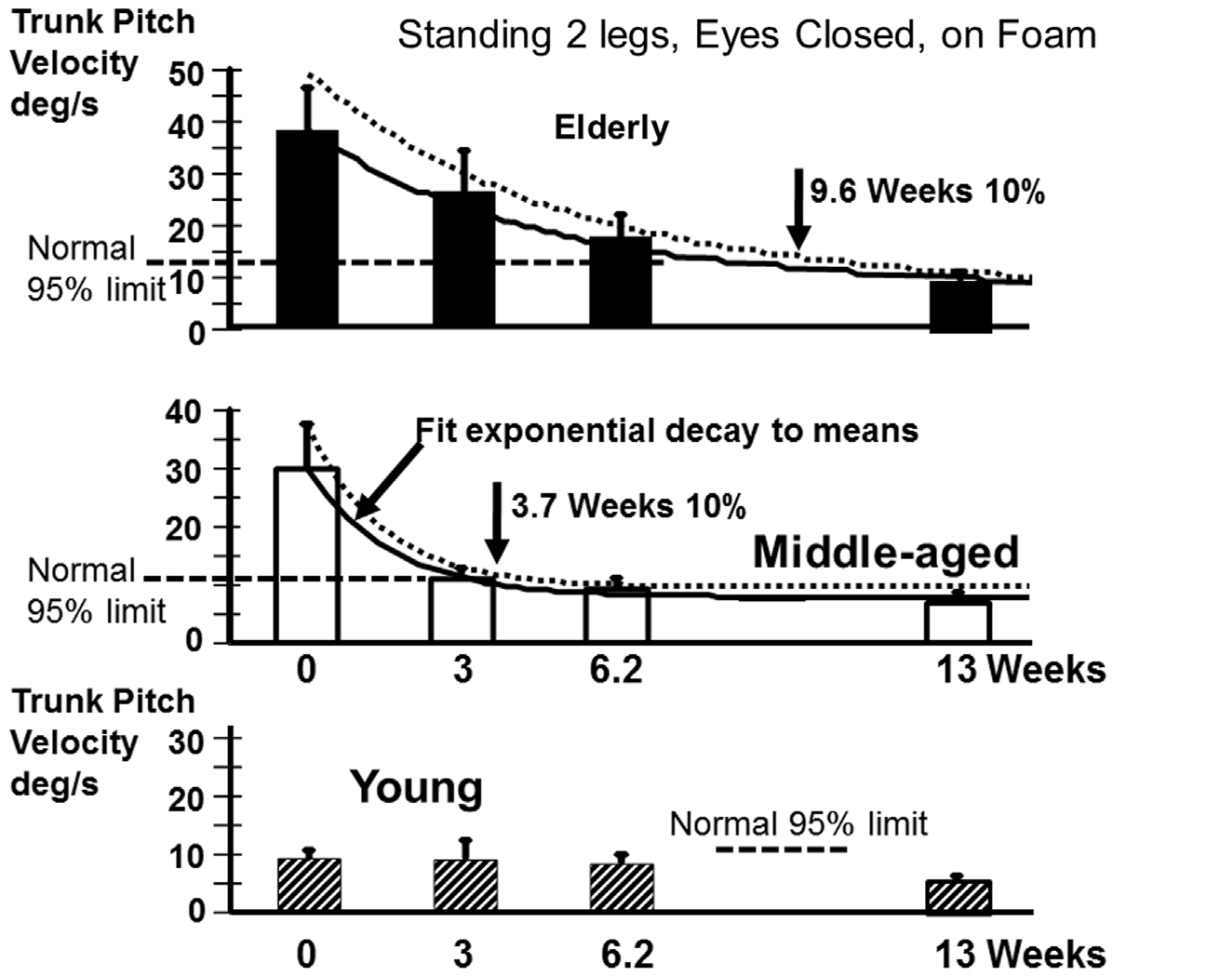
**Recovery time courses of pitch velocity for the different age populations for the task standing eyes closed on foam**. The column heights indicate mean values at aUVL onset (0) and 3, 6.2, and 13 weeks after onset. The vertical bars on the columns represent the SEM. The thick line joining the means is an exponential fit (see Materials and Methods) to the change in the mean value over time. The dashed line above the full line is an exponential fit (same model form) to the means plus the SEM. The recovery times to 10% of steady state are marked by vertical arrows in the figure. The upper 95% limit of normal sway for the age-group is marked by a dashed horizontal line. Note the recovery of the young was so rapid that no model fit was possible.

Recovery to the steady state level of stance balance control after aUVL was normally complete for all age-groups by 13 weeks (Figure 4). Recovery of the normal stance control was, however, slower in the elderly (as modeled by the exponential fit in Figure 4) taking approximately 10 weeks to reach within 10% of the steady state value. The middle-aged took less time, 3.7 weeks, to reach the 10% level. Recovery in the young was so rapid that an exponential fit to their time series data was not possible.

**Figure 5:**
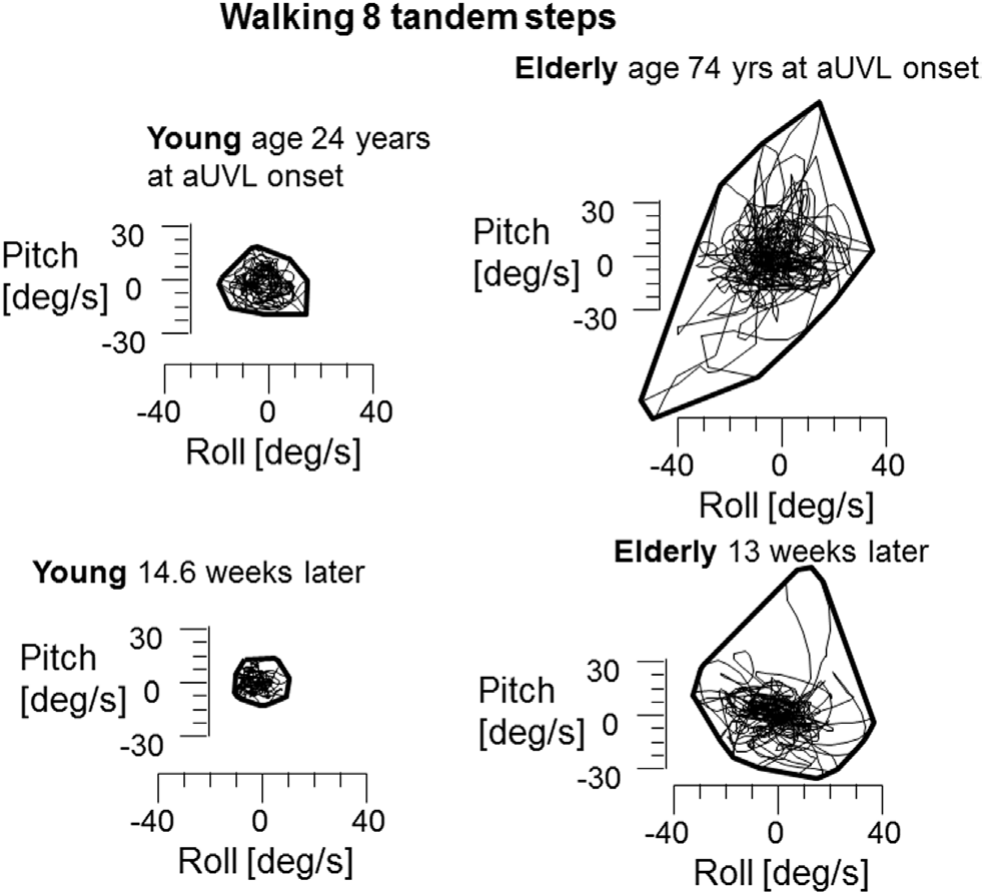
**Plots of roll versus pitch velocity for the task of walking eight tandem steps**. The layout of the figure is identical to that of Figure 2. The young and elderly patients in this example had no peripheral vestibular recovery. CP young 85% at onset to 95% after 13 weeks, elderly person 74% at onset to 100% at 13 weeks. Note the significantly larger sway at onset and at 13 weeks for the elderly person.

**Figure 6:**
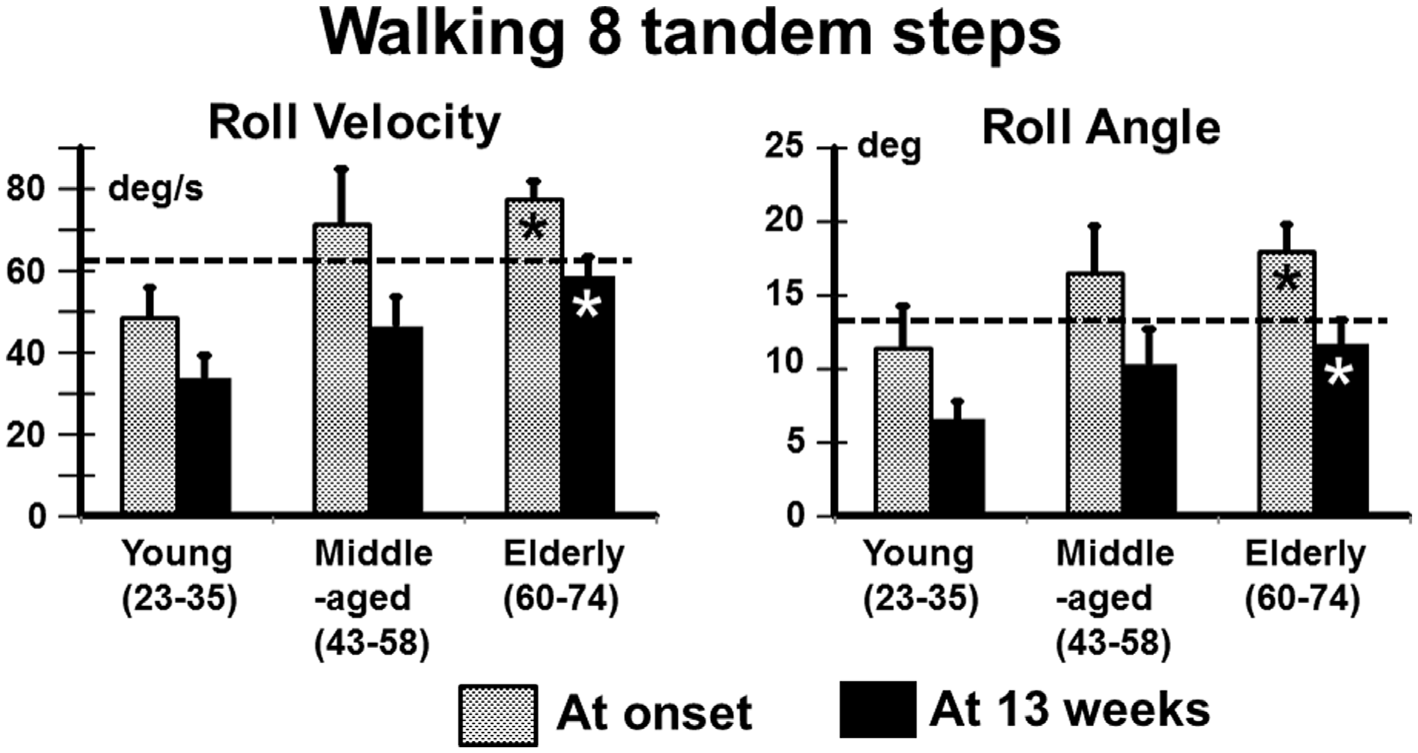
**Means and SEM of roll angular velocity and angle for the task of walking eight tandem steps in the different age-groups**. The layout of the figure is identical to that of Figure 1.

In contrast to stance, control of roll angle and angular velocity is more crucial than that of pitch when walking in tandem steps. The examples in Figure 5 (again patients with no peripheral recovery) and the mean column plots of Figure 6 indicate that these measures are significantly greater in the elderly than the young at onset and at 13 weeks (range of *p* values 0.007–0.045). In addition, the values for the elderly were significantly greater than values of healthy age-matched controls at onset and at 13 weeks (*p* < 0.006, see Table 1). There were no significant differences in balance improvement in the young and elderly between aUVL onset and 13 weeks for the tandem gait task (see differences in pairs of column plots in Figure 6).

**Figure 7:**
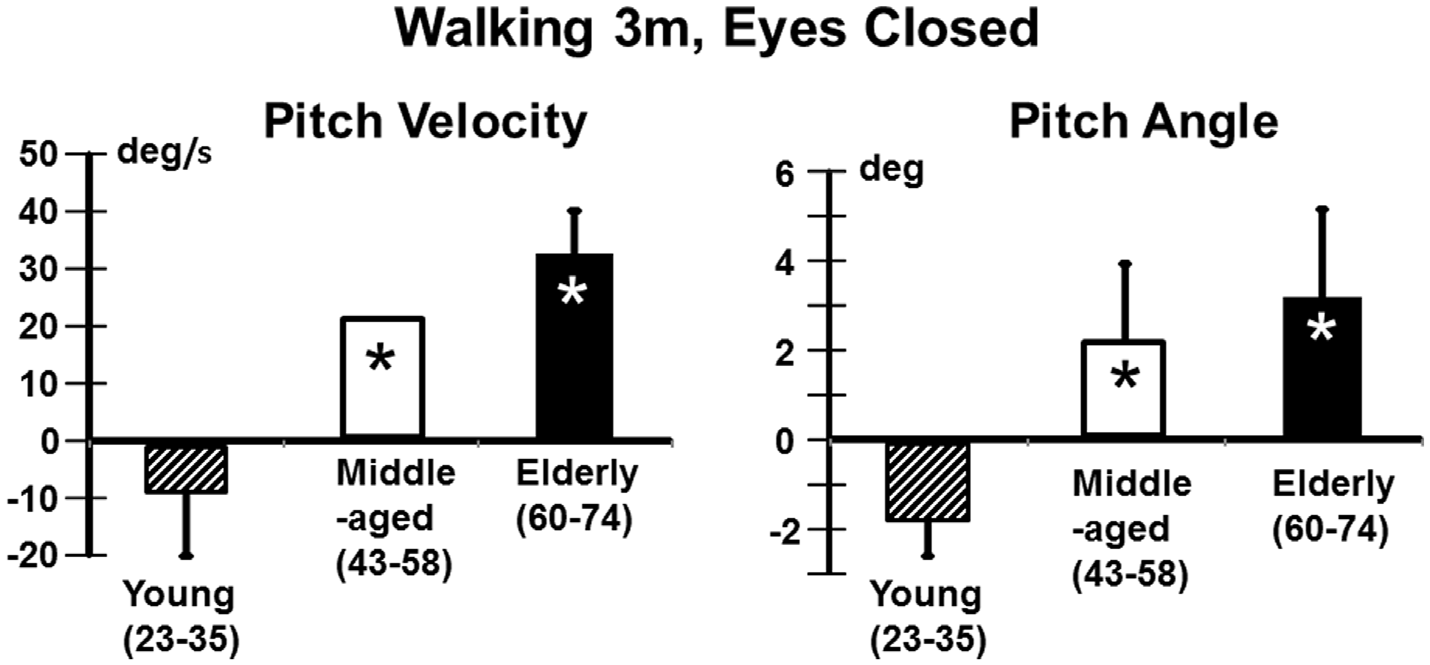
**Improvement in pitch angular velocity and pitch angle for the task of walking 3 m with eyes closed**. As indicated by the asterisks the improvement in the middle-aged and elderly is significantly greater than that of the young (*p* < 0.05). However, as indicated in Figure 8, trunk sway measures of the young are weakly affected by the aUVL.

**Figure 8:**
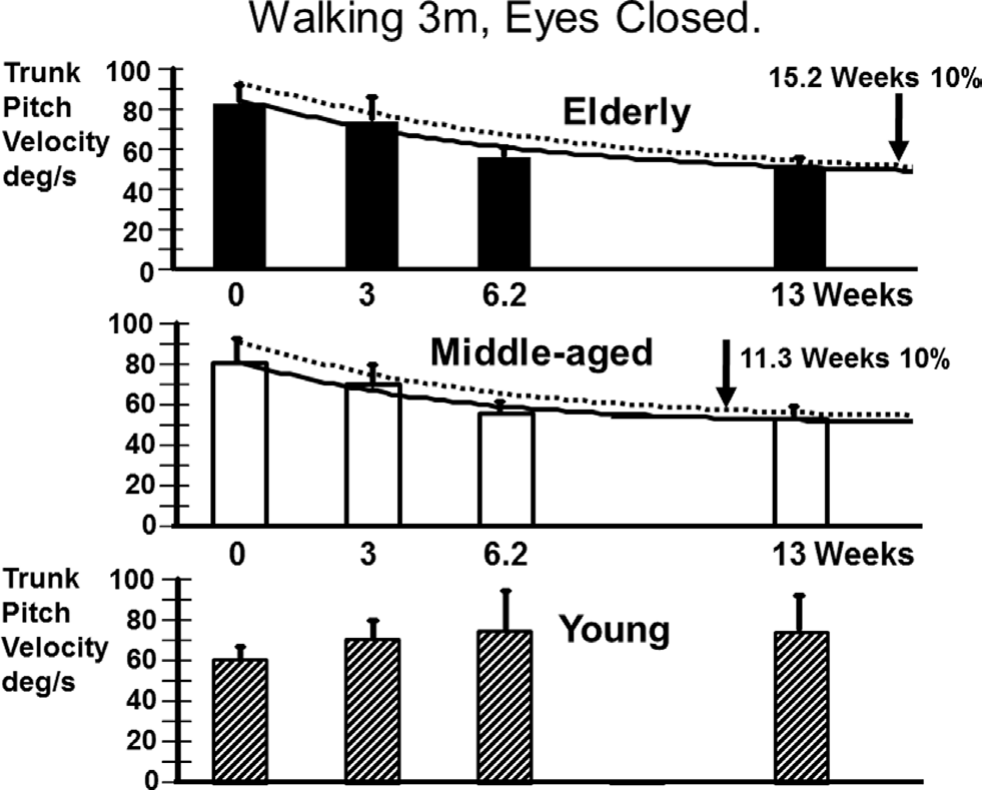
**Recovery time courses of pitch velocity for the different age populations for the task of walking 3 m with eyes closed**. The layout of the figure is identical to that of Figure 4. Note that the data of the young cannot be fitted by an exponential decay model.

We also examined the gait tasks walking 3 m while pitching the head up and down with eyes open, and walking without voluntary head movements but with eyes closed. The results for these tests were similar. The elderly had values outside of the range of healthy elderly at aUVL onset but generally not at 13 weeks (Table 1). Both pitch angle and velocity improvements were greater in the elderly (see Figure 7) for the walking eyes closed task when compared to the young (range of *p* values 0.004–0.03). This was due partly to the greater onset values of the elderly. However, this effect was partially counteracted by the slower recovery in the elderly. As shown in Figure 8, pitch velocity recovery was to within 10% of steady state at 15.2 weeks in the elderly and reached the same relative level at 11.3 weeks in the middle age. In contrast, the young increased pitch velocity over time for the eyes closed walking task. This was due to significant increase in gait speed (see Table 1). Both the young and the elderly had increased gait speed at 13 weeks for both of these gait tasks (Table 1).

## Discussion

Vestibular sensory inputs have a major influence on balance control even if this influence is not as powerful as that on the VOR (10, 11, 26, 29). Thus, it is to be expected that an aUVL initially has a profound effect on VOR and balance function. An intriguing point is that central compensation is able to restore VOR and balance function to nearly normal levels even when peripheral sensory recovery is absent (see Figures 1, 3 and 6). That is, within 13 weeks after an aUVL VOR function and balance control improve to approximately normal values (6, 7, 11). As VOR function and balance control (in the form of trunk sway) deteriorate with age (12–14, 16, 17, 19, 22), we had expected that there would be a detrimental effect of age on the recovery of both functions after aUVL. Surprisingly we found that for balance tasks, but not for VOR tasks, the elderly were more detrimentally influenced by aUVL than young subjects. VOR tests with low accelerations were the exception with a greater influence on the elderly. That is, with this minor exception, the combined effect of central compensation and peripheral recovery within VOR pathways after an aUVL was similar for the young and elderly.

The question arises whether the slightly decreased VOR values we observed for the elderly (see Figure 1) were the results of differences in the number of left and right deficits in each age-group. For example, vHIT gains are 15% larger for head impulses to the right compared to the left when the right eye is measured (24, 30). The number of right deficits ears was greater in the young compared to the elderly (61.5 versus 41.6%) and this difference might have influenced the results in the direction of trend observed (see Figure 1). However, a similar trend for larger deficit side responses in the young was also noted for the ROT results (see Figure 1). Therefore, we considered the greater number of right side deficits in the young not to have influenced our results.

The greater effect of the aUVL on balance control of the elderly (and to a lesser extent for the middle-aged) was manifest as a greater divergence from normal reference values at onset (Table 1) and in the time course of recovery (see Figures 4 and 8). In other words, the aUVL had a greater absolute effect on the balance control in the elderly. Also in contrast to our expectations, when differences occurred, the recovery was greater in the elderly, because balance control in the young was hardly changed from normal. Another factor influencing the differences in balance control improvement was the rate of recovery which was slower for the elderly. Thus, the elderly remained with worse balance control than the young over a longer period of time. Differences in improvement were pronounced in balance tests with vision absent, reinforcing data indicating that the visual influence on gait and stance is enhanced following vestibular loss (4, 11). Although proprioceptive influences are a major influence on balance control, these influences are not increased as much as visual influences following an aUVL (4).

When differences in recovery were present with age, more recovery was also seen in the pitch plane than in the roll plane. This supports the idea that trunk pitch and trunk roll control are controlled in a different way by the central nervous system (29). This finding also suggests that recovery within the pathways responsible for pitch plane control is faster and more effective than the recovery within the pathways for roll plane control (4). Another factor influencing the faster recovery in the pitch plane could be the larger number of degrees of freedom for the roll compared to pitch plane and the use of a stiffening strategy in the elderly (31). Stiffening leads to balance instability particularly in the roll plane (32).

There was not a systematic change in durations for gait trials in the young with respect to elderly subjects. All subjects reduced gait task durations (increased gait speed) in the weeks after aUVL onset. However, the young increased pitch trunk sway velocity as expected with increases in gait speed [see Figure 8 (17)]. In contrast, the middle-aged and elderly decreased sway as gait speed increased over time. Thus, the presence of central compensation for a vestibular loss reducing trunk sway appears to counteract the normal increase in trunk sway with increased gait speed in those over 35 years of age. Therefore, it is an open question whether elderly subjects would have improved more if we had asked them to walk faster. Brandt et al. (5) showed that elderly aUVL subjects were better of running than walking following on aUVL.

Differences with age on the recovery of VOR function were basically not observed, except at very low accelerations during ROT tests at 5°/s^2^ but major age-related differences in balance control were observed. These findings support our previous observations that VOR and balance test measures are either weakly or not correlated (4, 11). The strongest correlation is negative between the visual contribution to stance control and the deficit side response amplitude for 20°/s^2^ ROT (*R* = 0.48). Other correlations are lower than *R* = 0.4 (4). It should be emphasized that peripheral recovery as shown by caloric testing did not show any difference between the age-groups. On average, recovery was much less than 100% in each age-group. CP values reduced from 87 to 52% (see Figure 1), on average, without differences between age-groups, even though more of the elderly had a lack of peripheral recovery and insufficient VOR compensation at 13 weeks. In general, both young and elderly had VOR measures that were equally different from those of healthy controls. These results support our previous findings that lack of VOR recovery does not imply that balance function has not recovered and, oppositely, the recovery in VOR measures does not imply a recovery in balance function (4, 11). Rather, the young whose VOR values were pathological after an aUVL had balance control that was hardly changed from normal by the UVL, whereas the elderly and to a lesser extent the middle-aged had ongoing stance and gait balance deficits for several weeks after an aUVL despite significant improvements in VOR measures.

Despite the lack of strong correlations noted above, it is possible that some similar processes underlie VOR and VSR recovery following an aUVL. For example, a lack of peripheral vestibular recovery (as indicated by the greater number of elderly patients with caloric CP values greater than 90% at 13 weeks) and reduced central compensation (as determined by ROT responses of the elderly) may have a correlated negative influence on the central compensation for balance deficits. To answer this question in detail, additional studies with larger numbers of patients are required. Regardless of the cause, the longer period of gait deficits after aUVL in the elderly implies that the physical therapy needs of the elderly should be focused initially on stance and gait balance control and then after 6–8 weeks primarily on gait balance control, even if ROT and vHIT VOR tests indicate a return to normal function during this period.

The elderly are presumably always adapting to the constant worsening in balance control with aging over the age of 60 years (18). Nonetheless, they were unable to cope with the sudden unstable balance caused by an aUVL. To improve their responses to an aUVL those with risk factors indicative of UVL [e.g., see Chuang et al. (33) could be treated with physical therapy ahead of a possible UVL just as has been employed prior to surgical removal of cerebellar pontine angle tumors (34)]. In fact, it is one of the weaknesses of the current study that we were not able to control the activity levels of the patients prior to an aUVL nor for their adherence to prescribed physical therapy post aUVL.

## Conclusion

Our results indicate that aUVL due, presumably, to vestibular neuritis causes a relative worsening of stance and gait balance control in the elderly compared to the young. At acute onset, the elderly are more unstable than the young and take longer to reacquire the balance abilities of age-matched controls. VOR function in the young may fail to improve to normal levels rapidly after aUVL onset; however, this appears not to prevent rapid recovery of normal balance control in the young.

## Author Contributions

JA conceived the experimental design, planned the data collection, constructed the figures, and rewrote drafts of the manuscript. AS helped collect data, worked on the statistics, and wrote the first drafts of the manuscript. DT also worked on early drafts of the paper and helped collect data. FH was involved in setting up the experimental design (clinical tests), conceived the modelling procedures, and worked on data analysis.

## Conflict of Interest Statement

The authors Flurin Honegger and John H. J. Allum declare a conflict of interest as they both worked as consultants for the company producing the SwayStar equipment used in this study. The remaining authors declare that the research was conducted in the absence of any commercial or financial relationships that could be construed as a potential conflict of interest.

## References

[1] Lacour M. Restoration of vestibular function: basic aspects and practical advances for rehabilitation. Curr Med Res Opin (2006) 22(9):1651–9. 10.1185/030079906X115694 16968568

[2] Horak FB. Postural compensation for vestibular loss and implications for rehabilitation. Restor Neurol Neurosci (2010) 28(1):57–68. 10.3233/RNN-2010-0515 20086283 PMC2965039

[3] Allum JH. Recovery of vestibular ocular reflex function and balance control after a unilateral peripheral vestibular deficit. Front Neurol (2012) 3:83. 10.3389/fneur.2012.00083 22623921 PMC3353232

[4] Allum JHJ Honegger F Recovery times of stance and gait balance control after an acute unilateral peripheral vestibular deficit. J Vestib Res (2015) 25:219–31. 10.3233/VES-150561 26890423

[5] Brandt T Strupp M Benson J. You are better off running than walking with acute vestibulopathy. Lancet (1999) 354(9180):746. 10.1016/S0140-6736(99)03179-7 10475195

[6] Allum JHJ Ledin T Recovery of vestibulo-ocular function in subjects with acute peripheral vestibular loss. J Vestib Res (1999) 9:135–44. 10378185

[7] Allum JH Adkin AL. Improvements in trunk sway observed for stance and gait tasks during recovery from an acute unilateral peripheral vestibular deficit. Audiol Neurootol (2003) 8(5):286–302. 10.1159/000071999 12904683

[8] De Waele C Graf W Josset P Vidal PP. A radiological analysis of the postural syndromes following hemilabyrinthectomy and selective canal and otolith lesions in the guinea pig. Exp Brain Res (1989) 77(1):166–82. 10.1007/BF00250579 2792260

[9] Halmagyi GM Weber KP Curthoys IS. Vestibular function after acute vestibular neuritis. Restor Neurol Neurosci (2010) 28(1):37–46. 10.3233/RNN-2010-0533 20086281

[10] MacDougall HG Curthoys IS. Plasticity during vestibular compensation: the role of saccades. Front Neurol (2012) 3:21. 10.3389/fneur.2012.00021 22403569 PMC3289127

[11] Allum JHJ Honegger F. Relation between head impulse tests, rotating chair tests, and stance and gait posturography after an acute unilateral peripheral vestibular deficit. Otol Neurootol (2013) 34(6):980–9. 10.1097/MAO.0b013e31829ce5ec 23820798

[12] Iwasaki S Yamasoba T Dizziness and imbalance in the elderly: age-related decline in the vestibular system. Aging Dis (2015) 6(1):38–47. 10.14336/AD.2014.0128 25657851 PMC4306472

[13] Baloh RW Enrietto J Jacobson KM Lin A. Age-related changes in vestibular function: a longitudinal study. Ann N Y Acad Sci (2001) 942:210–9. 10.1111/j.1749-6632.2001.tb03747.x 11710463

[14] Barin K Dodson EE. Dizziness in the elderly. Otolaryngol Clin North Am (2011) 44(2):437–54,x. 10.1016/j.otc.2011.01.013 21474016

[15] Hsieh LC Lin HC Lee GS. Aging of vestibular function evaluated using correlational vestibular autorotation test. Clin Interv Aging (2014) 9:1463–9. 10.2147/CIA.S67720 25214774 PMC4159125

[16] Gill J Allum JH Carpenter MG Held-Ziolkowska M Adkin AL Honegger F Trunk sway measures of postural stability during clinical balance tests: effects of age. J Gerontol A Biol Sci Med Sci (2001) 56(7):M438–47. 10.1093/gerona/56.7.M438 11445603

[17] Goutier KM Jansen SL Horlings CG Kung UM Allum JH The influence of walking speed and gender on trunk sway for the healthy young and older adults. Age Ageing (2010) 39(5):647–50. 10.1093/ageing/afq066 20558480

[18] Hegeman J Shapkova EY Honegger F Allum JHJ. Effect of age and height on trunk sway during stance and gait. J Vestib Res (2007) 17(2–3):75–87. 18413900

[19] Peterka RJ Black FO. Age-related changes in human posture control: sensory organization tests. J Vestib Res (1990) 1(1):73–85. 1670139

[20] Baloh RW Ying SH Jacobson KM. A longitudinal study of gait and balance dysfunction in normal older people. Arch Neurol (2003) 60(6):835–9. 10.1001/archneur.60.6.835 12810488

[21] Rauch SD Velazquez-Villaseñor L Dimitri PS Merchant SN. Decreasing hair cell counts in aging humans. Ann N Y Acad Sci (2001) 942(1):220–7. 10.1111/j.1749-6632.2001.tb03748.x 11710464

[22] Peterka RJ Black FO Schoenhoff MB. Age-related changes in human vestibulo-ocular reflexes: sinusoidal rotation and caloric tests. J Vestib Res (1990) 1(1):49–59. 1670137

[23] Mossman B Mossman S Purdie G Schneider E. Age dependent normal horizontal VOR gain of head impulse test as measured with video-oculography. J Otolaryngol Head Neck Surg (2015) 44(1):29. 10.1186/s40463-015-0081-7 26141721 PMC4506627

[24] McGarvie LA MacDougall HG Halmagyi GM Burgess AM Weber KP Curthoys IS. The video head impulse test (vHIT) of semicircular canal function – age-dependent normative values of VOR gain in healthy subjects. Front Neurol (2015) 6:154. 10.3389/fneur.2015.00154 26217301 PMC4495346

[25] Allum JHJ Honegger F Ura M. Documentation of the recovery course and deficit side localization of an acute unilateral vestibular deficit using four-quadrant diagrams of slow phase velocity. Acta Otolaryngol Suppl (1991) 481:419–23. 10.3109/00016489109131436 1927431

[26] MacDougall HG Weber KP McGarvie LA Halmagyi GM Curthoys IS. The video head impulse test: diagnostic accuracy in peripheral vestibulopathy. Neurology (2009) 73(14):1134–41. 10.1212/WNL.0b013e3181bacf85 19805730 PMC2890997

[27] Cleworth T Carpenter MG Allum JHJ, editors. Differences in head impluse test results due to analysis techniques. Abstract for ADANO Conference; Bern (2015). 10.3233/VES-170614 29064828

[28] Allum JH Ledin T. Recovery of vestibulo-ocular reflex-function in subjects with an acute unilateral peripheral vestibular deficit. J Vestib Res (1999) 9(2):135–44. 10378185

[29] Carpenter MG Allum JH Honegger F. Vestibular influences on human postural control in combinations of pitch and roll planes reveal differences in spatiotemporal processing. Exp Brain Res (2001) 140(1):95–111. 10.1007/s002210100802 11500802

[30] Weber KP Aw ST Todd MJ McGarvie LA Pratap S Curthoys IS Chapter 3.7 – Inter-ocular differences of the horizontal vestibulo-ocular reflex during impulsive testing. In: Christopher K Leigh RJ, editors. Progress in Brain Research. (Vol. 171), Amsterdam: Elsevier (2008). p. 195–8. 10.1016/S0079-6123(08)00626-2 18718300

[31] Allum JHJ Carpenter MG Honegger F Adkin AL Bloem BR. Age-dependent variations in the directional sensitivity of balance corrections and compensatory arm movements in man. J Physiol (2002) 542(Pt 2):643–63. 10.1113/jphysiol.2001.015644 12122159 PMC2290411

[32] Grüneberg C Bloem BR Honegger F Allum JHJ. The influence of artificially increased hip and trunk stiffness on balance control in man. Exp Brain Res (2004) 157(4):472–85. 15138751 10.1007/s00221-004-1861-x

[33] Chuang Y-M Chern C-M Liao W-H Hsu L-C Lien C-F Lirng J-F Comorbid intracranial vertebral artery asymmetry as a risk factor for severe vestibular neuronitis. Otol Neurotol (2011) 32(3):478–82. 10.1097/MAO.0b013e31820e785c 21317672

[34] Magnusson M Karlberg M Tjernström F ‘PREHAB’: vestibular prehabilitation to ameliorate the effect of a sudden vestibular loss. NeuroRehabilitation (2011) 29(2):153–6. 10.3233/NRE-2011-0689 22027076

